# In Memoriam—William Newton Morrison, D. D. S

**Published:** 1897-02

**Authors:** 


### OBITUARY.

### In Memoriam.

WILLIAM NEWTON MORRISON, D.D.S.

A special meeting of the St. Louis Dental Society was held on December 22, 1896. President F. F. Fletcher opened the meeting with the following remarks:

*Members of the St. Louis Dental Society:*—It was with heavy hearts that your officers sent you notice to meet here in special session this evening. As lightning from a clear sky came the news to us yesterday morning that one of our oldest and most respected and esteemed members lay cold in death.

A man who, but one short week ago sat in his place in the councils of this body and took part in the deliberations, and whom we had every reason to hope and expect would meet with us for years and aid us with his counsel and advice.

A man known and respected wherever dentistry is practiced. He was a carefnl student, a ripe scholar and an inventor of much ability.

Few men in our profession have been more progressive, or lived to see their experimental work in untried fields adopted and approved by all. He was a pioneer in crown-work, bridge-work and implantation; they stand to-day his most lasting monument. But he is gone. The last page of his life is before us.

My friends, in the death of William N. Morrison the dental world loses one of its pioneers and brightest stars. This society has lost one of its ablest men and most staunch supporters.

Every member has lost a friend whose place will not easily be filled. May no uncharitable word be spoken, but as we say peace to his ashes may his memory ever be kept green by the greatness of his achievements.

Appropriate remarke were made by Drs. H. J. McKellops, G. A. Bowman, J. H. Kennerly and Wm. Conrad.

The following committee, Drs. H. J. McKellops, John G. Harper and A. H. Fuller, was appointed to prepare a biographical sketch of Dr. Morrison. The committee, on January 5th, presented this report.

BIOGRAPHICAL.

William Newton Morrison, D.D.S., was born at East Springfield, Ohio, May 25, 1842, and died at Hot Springs, Arkansas, December 20, 1896.

He was one of thirteen children of John R. Morrison. Those surviving are James B., Mrs. Lane, of Kansas City, Mo., and Mrs. Cook, of Mendota, Ill.

Dr. Morrison's early education was but meager, obtained in the common schools. He worked in his father's saw-mill while at home, but left in 1858; came to St. Louis to become a student of his brother James B., the inventor of the Morrison Dental Engine and Chair.

He arrived at his brother's office penniless, having spent his last money to have his boots blackened and his clothes brushed so as to make a presentable appearance. The brothers kept bachelor's hall in the same building occupied as an office.

James B. went to Europe in 1862, and William took charge of Dr. H. J. McKellops' office, who also left the city about that time.

In 1864 he graduated from the Ohio Dental College.

In 1868 he and Miss Cornelia Holme, of Hannibal, Mo., were married. Two sons were added to the family, Peter Holme and William N., the former is married and has a son two years old.

The doctor was so successful in his early practice as to be able in 1872 to build at 1401 Washington Avenue, a house which combined a dwelling and a dental office, each complete for the purpose intended, the plans being made by the doctor and published with illustrations in the *Missouri Dental Journal.*

Dr. Morrison belonged to a family of dentists, having an uncle, two brothers and two cousins who followed that profession.

As a dentist Dr. Morrison kept abreast of the prfossion and was one of the first to use the mallet and to construct gold crowns.

He was one of the first to revive the "planting" of teeth, as he called it, his first cases being reported in 1874. The last report was made at the last meeting of the St. Louis Dental Society he attended, on December 1, 1896.

He was fond of visiting the offices of dentists, and when in a town or city made it a rule to call on the members of the profession there located, to learn all he could and cheerfully give to others ideas which he thought might interest or benefit them. He always took pleasure in entertaining dentists of the city and those from abroad.

He became a Mason in 1865. He was brought up a Methodist, but after marrying joined the Presbyterian Church, belonging to the Second.

Dr. Morrison was a constant attendant of dental societies, and belonged to the American, Southern, Mississippi Valley,

Illinois, Missouri and St. Louis, frequently writing papers, giving clinics and joining in the discussions, also holding office in those of which he was an active member, having been president of the Missouri and St. Louis, twice of the latter. He took an active part in the Missouri Dental College, filling the chair of Mechanical Dentistry, also acted as demonstrator, and gave clinics every session when in the city.

Dr. Morrison traveled extensively for a dentist. In July, 1878, he started on a trip around the world which consumed about a year. While on this trip he learned all he could regarding the status of the profession in the counties visited, bringing home with him specimens of work found while abroad. He also made a large collection of photographs of places visited. These he frequently publicly exhibited in the aid of charity. In 1890 he took a trip to Germany for his health, again in 1894, accompanied by his wife, he made a trip to Europe. He has been to the West Indies, and also traveled extensively in this country.

Dr. Morrison was a writer for our journals, and many of his articles and items are to be found in the *Missouri Dental Journal* and its successor, the *Archives of Dentistry*, both of which he aided in more ways than one. Of the former, he was one of the editors of the department of Mechanical Dentistry for four years.

Dr. Morrison was a public-spirited citizen and did his share for the public good; he, at his own expense, placed numbers on the streets on Washington Avenue, from Jefferson Avenue to King's Highway.

Dr. Morrison was as well-known and esteemed in this country and abroad as any dentist in our city. He was the inventor of the Morrison Dental Bracket, being one of the first put on the market.

Dr. Morrison made friends wherever he went, and none ever heard him say aught against any one.

His success was gained by hard, faithful work, and he was ever ready to lend a helping hand to his fellowman.

H. J. McKellops,

John G. Harper,  } Committee.

A. H. Fuller,

